# Glaucoma: Current and New Therapeutic Approaches

**DOI:** 10.3390/biomedicines12092000

**Published:** 2024-09-03

**Authors:** Hsin-Pei Lee, Ta-Hsin Tsung, Yu-Chien Tsai, Yi-Hao Chen, Da-Wen Lu

**Affiliations:** 1 Department of Ophthalmology, Tri-Service General Hospital, National Defense Medical Center, Taipei 114, Taiwan; 2 Department of Ophthalmology, Taoyuan Armed Forces General Hospital, Taoyuan 325, Taiwan

**Keywords:** glaucoma therapeutic approaches, neuroprotection, neurodegeneration, oxidative stress, neuroinflammation, excitotoxicity

## Abstract

Glaucoma is identified by the loss of retinal ganglion cells (RGCs). The primary approach to managing glaucoma is to control intraocular pressure (IOP). Lately, there has been an increasing focus on neuroprotective therapies for glaucoma because of the limited effectiveness of standard methods in reducing IOP and preventing ongoing vision deterioration in certain glaucoma patients. Various drug-based techniques with neuroprotective properties have demonstrated the ability to decrease the mortality of retinal ganglion cells. This study will analyze the currently recommended drug-based techniques for neuroprotection in the prospective treatment of glaucoma.

## 1. Introduction

Glaucoma is a group of long-lasting and worsening optic neuropathies that are identified by the reduction of retinal ganglion cells and the consequent problems with vision [1]. Despite the current lack of complete understanding regarding the precise etiology of the disease, it has been firmly established that altering intraocular pressure (IOP) represents the sole adjustable risk factor for mitigating the potential visual impairment associated with glaucoma. The primary approach for managing adult glaucoma is through medical care, which has shown significant and rapid breakthroughs in therapeutic interventions for this illness [2]. Lately, there has been an increasing focus on retinal ganglion cells (RGCs) and the specific processes that make them vulnerable to damage and deterioration in the retina and optic nerve. Additionally, there is progress being made in developing medications that protect the nervous system from damage caused by glaucoma. These treatments could be employed in addition to ocular hypotensive methods to better preserve RGCs and minimize visual loss [3,4].

## 2. Present IOP-Reducing Medications

In the management of glaucoma, drugs that reduce IOP play a pivotal role. The selection of medication is contingent upon the individual patient’s unique requirements, the precise classification of glaucoma, and any existing contraindications, necessitating constant surveillance to ascertain the efficacy of the treatment and mitigate potential adverse reactions.

### 2.1. Beta Adrenergic Antagonists (β-Blockers)

The identification of Beta-adrenergic antagonists (β-blockers) as a potential therapeutic approach for glaucoma was fortuitous and sprang from cardiovascular investigations. During the late 1960s and early 1970s, β-blockers were predominantly formulated and employed for the treatment of cardiovascular ailments such as hypertension and arrhythmias [5]. Researchers noted that these drugs also influenced IOP, prompting additional investigations into their potential applications in ophthalmology. They prevent sympathetic nerve terminals in the ciliary body’s epithelium from functioning, and blocking adrenergic β-receptors in the ciliary body leads to a reduction of the generation of aqueous humor. This inhibition results in a reduction of the synthesis of aqueous humor. β-blockers can be categorized into two primary categories: nonselective and cardioselective, sometimes known as β1 selective. Individuals with asthma and chronic obstructive pulmonary disease (COPD) have been found to exhibit a higher level of tolerance towards the latter category. β-blockers offer various benefits, such as their cost-effectiveness and the simplicity of once-daily administration [6]. Research findings indicate that timolol, a β-blocker commonly used, possesses the capacity to substantially diminish IOP by approximately 20–35% [7]. It emerged as the primary medical therapy for glaucoma and ocular hypertension (OHT) and continued to be the unquestioned first option until the prostaglandin analogs were introduced in 1996 [8].

### 2.2. Adrenergic Agonists

Adrenergic agonists work by inhibiting the synthesis of aqueous humor and enhancing the drainage of uveoscleral fluid from the eye. In practical settings, selective drugs have largely replaced nonselective adrenergic agonists. Currently, brimonidine, which was introduced in the mid-1990s, stands out as the sole medication utilized for long-term treatments in this category. Several studies have shown that giving alpha-agonists can change the amounts and activities of matrix metalloproteinases and the tissue inhibitors that go with them. These proteins have a significant impact on the degradation of the extracellular matrix, which makes it possible for more fluid to flow through the uveoscleral pathway [9].

A smaller-scale randomized controlled trial was performed to see how brimonidine treatment affected the sensitivity of contrast in people who had just been diagnosed with glaucoma. The results of this study demonstrated that those who received brimonidine exhibited a statistically significant enhancement in contrast sensitivity compared to those who were administered timolol. The aforementioned observation, regardless of any drop in intraocular pressure, implies the existence of a neuroprotective mechanism in glaucoma [10,11].

Nevertheless, it is advisable to refrain from utilizing alpha2-adrenergic agonists for topical therapy in pediatric patients below the age of 12, owing to the potential for significant adverse reactions. In certain instances, these deleterious consequences can be highly severe, potentially resulting in a state of unconsciousness in infants and toddlers.

### 2.3. Carbonic Anhydrase Inhibitors (CAIs)

Carbonic anhydrase inhibitors (CAIs) function by suppressing the activity of the enzyme carbonic anhydrase. This leads to a decrease in the secretion of bicarbonate and aqueous humor. Medications with a significant historical lineage have been used to treat open-angle glaucoma (OAG). Systemic CAIs, such as acetazolamide and methazolamide, have demonstrated efficacy in decreasing IOP in patients with glaucoma with reports demonstrating the efficacy of acetazolamide in reducing IOP as early as 1954. However, their administration is linked to notable adverse effects, including fatigue, abnormal sensations, nausea, dizziness, hypokalemia, nephrolithiasis, and weight loss. Stevens-Johnson syndrome, toxic epidermal necrolysis, and aplastic anemia are infrequent yet severe consequences that may manifest [12,13].

The development of topical CAIs, like dorzolamide (Trusopt) and brinzolamide (Azopt), has been focused on achieving localized administration in order to minimize the occurrence of systemic side effects. These pharmaceuticals have subsequently emerged as a noteworthy classification, including easily obtainable formulations that have been formulated in conjunction with other prescribed drugs to mitigate IOP [14].

Dorzolamide–timolol (Cosopt, FDA authorized in 1998) and brinzolamide–brimonidine (Simbrinza, FDA approved in 2013) are combination medicines that are commonly used to reduce IOP and enhance patient adherence [15]. Switching from a combination of four drugs to a combination of latanoprost–timolol and brinzolamide–brimonidine resulted in significant improvements in IOP and patient adherence. A further study demonstrated that the combination of brinzolamide–brimonidine with a prostaglandin analogue resulted in a more effective reduction of IOP compared to using a prostaglandin analogue alone [16]. Both the combination therapy of brinzolamide–brimonidine and dorzolamide–timolol, when provided as a single drop, were found to be equally effective as each of the individual component medicines when administered separately [14]. CAIs may be employed when second-line beta blockers are contraindicated, although they are frequently more effective when prescribed in conjunction with a beta-blocker [17].

### 2.4. Prostaglandin Analogues (PGAs)

Prostaglandin analogues (PGAs), including tafluprost, latanoprost, travoprost, bimatoprost, and omidenepag isopropyl, have shown to be more effective at managing IOP. Following this, the pursuit of carbonic anhydrase inhibitors, β-blockers, and α-2 agonists is observed in the literature, as indicated by previous research studies [18,19].

Five classes of prostaglandins exist: prostaglandin E2 (PGE2), F2 (PGF2), I2 (PGI2), D2 (PGD2), and thromboxane A (TXA2). The utilization of PGF2 and prostaglandin FP agonists has resulted in a reduction of IOP. In order to achieve this particular impact, the uveoscleral outflow is augmented in an atypical manner. This process can also be achieved by activating prostaglandin receptors located in the ciliary muscle, iris root, and sclera by relaxing the smooth muscle, modifying the cytoskeleton, and remodeling the extracellular matrix in the uveoscleral pathway [20,21]. Furthermore, it could potentially improve the outflow of aqueous humor by activating the FP receptors found in the trabecular meshwork.

PGAs are the most effective medications for lowering intraocular pressure (IOP), followed by β-blockers, α-2 agonists, and carbonic anhydrase inhibitors [22]. Several studies have provided evidence indicating that latanoprost (FDA approved in 1996) demonstrates enhanced effectiveness in the reduction of IOP [21,23] and that bimatoprost exhibits greater efficacy in the reduction of IOP in comparison to latanoprost [24]. Nevertheless, there have been different research outcomes that indicate similar efficacy between the two drugs [25].

Adverse effects on the eyes may include redness of the conjunctiva, increased growth of eyelashes, and various forms of pain such as eye irritation, itching, tears, a sensation of a foreign object in the eye, the formation of cysts in the iris, swelling of the macula, inflammation of the front part of the eye, and the reactivation of herpes simplex keratitis [26]. PGAs have a very safe profile when it comes to systemic side effects. These drugs are non-toxic to the circulatory and respiratory systems and have no negative side effects [27].

### 2.5. Muscarinic Receptor Agonist

Activation of muscarinic receptors in the ciliary muscle leads to an increased outflow of aqueous humor from the eye. The duration of this impact normally ranges from three to five hours. Hence, the administration of pilocarpine eye drops necessitates a minimum of four daily doses, posing challenges for patients in adhering continuously to the recommended therapeutic regimen [28]. Moreover, the administration of pilocarpine has the potential to elicit extensive parasympathetic reactions, including bronchospasm, heightened salivation, gastrointestinal disturbances, perspiration, and bradycardia, as a result of its activation of the cholinergic system [29].

### 2.6. Hyperosmotic Agent

Hyperosmotic drugs function by diminishing the quantity of aqueous fluid present in the ocular cavity, hence leading to a reduction in IOP. These medications are commonly employed in urgent scenarios or as a preoperative measure to transiently reduce IOP [30]. This category of pharmaceuticals encompasses orally delivered substances such as glycerin, isosorbide, and mannitol, which is supplied intravenously.

### 2.7. Rho Kinase Inhibitor

Rho kinase (ROCK) is a serine/threonine protein kinase that serves as an important downstream mediator of Rho signaling [31,32,33]. It reduces the density of actin stress fibers, which directly affects the cytoskeleton of the trabecular meshwork and Schlemm’s canal and improves the outflow facility. This differs from the intended goal of other glaucoma medications. Investigation into Rho kinase commenced in the late 1990s and has persisted up to the present day. Recent studies have produced a wealth of information regarding the various possible therapeutic applications of ROCK inhibitors in treating glaucoma, namely in maintaining IOP [34,35,36,37] and in treating neurodegenerative disease [38]. There is a limited amount of research examining the therapeutic impact of a Rho kinase inhibitor on diabetic retinopathy and its ability to promote repair in the corneal endothelium. In 2014, Ripasudil was approved in Japan for the particular treatment of ocular hypertension and glaucoma. Then, the efficacy of Rho-kinase inhibitors as anti-glaucoma drugs was validated with the release of Netarsudil in 2017. This drug was licensed in the United States in a 0.02% formulation [39]. Rhopressa, a Rho kinase inhibiting drug consisting of Netarsudil, gained FDA approval in December, 2017 [40]. Currently, ROCK inhibitors are primarily used in combination with other primary drugs for glaucoma treatment to guarantee a sufficient reduction in IOP [41].

### 2.8. Fixed Combinations 

Regarding the treatment of ocular hypertension and glaucoma, fixed combinations of medications that decrease IOP are now increasingly being used. These combinations provide various potential benefits compared to using the individual medications separately, such as increased convenience, enhanced adherence, reduced exposure to preservatives, and potential cost savings [42]. The current meta-analysis revealed that the utilization of fixed combinations with timolol, specifically dorzolamide/timolol, brinzolamide/timolol, and brimonidine/timolol, can lead to a reduction in IOP of over 30% [43,44,45].

## 3. Beyond IOP: Mechanisms of Neurodegeneration in Glaucoma

Neuroprotective treatments for glaucoma can help avoid the death of retinal ganglion cells and damage to the optic nerve by reducing ischemia and oxidative damage. While IOP is the sole identified variable that can be changed to lower the risk of glaucoma [46], emerging research indicates that injury to the optic nerve can persist even when IOP is effectively reduced [47,48,49]. There is a tremendous therapeutic need for neuroprotective methods that especially focus on the retinal ganglion cell and the underlying neurodegenerative processes [50,51].

### 3.1. Neurodegenerative Processes

Similar to other diseases, glaucoma exhibits an increased prevalence with advancing age, advances gradually and subtly, and is influenced by genetic factors [52]. A retrospective study revealed that patients with primary open-angle glaucoma (POAG) were more likely to acquire Alzheimer’s disease (AD) compared to people without POAG [53]. There is also increasing evidence that glaucomatous neuropathy affects not just the retina, but also the central nervous system (CNS) [54,55,56,57,58,59].

### 3.2. Oxidative Stress

Inflammation, hypoxia, ischemia, and mitochondrial malfunction can induce heightened oxidative stress, which can lead to glaucoma. The presence of mitochondrial damage and loss in the trabecular meshwork (TM) leads to both degenerative and apoptotic processes, ultimately resulting in cell loss [60]. Oxidative stress can directly damage RGCs as well [61,62]. Increasing evidence suggests that oxidative stress is a significant factor in the neuronal cell death that happens during glaucomatous neurodegeneration [63]. This oxidative stress impairs cellular homeostasis and disrupts normal cellular functions, contributing to a cascade of detrimental effects on the optic nerve and retinal tissues. Additionally, oxidative stress exacerbates the inflammatory response, further aggravating tissue damage and promoting the progression of glaucoma. Therefore, researchers are exploring antioxidant therapies and protective agents as a potential strategy to target oxidative stress, mitigate the progression of glaucomatous damage, and preserve visual function.

### 3.3. Neuroinflammation

Neuroinflammation is now recognized as a crucial role in the development of glaucoma due to the participation of immune and glial cells in the early stages of the illness. Astrocytes, microglia, and invading monocytes play important roles in the neuroinflammatory process in glaucoma [64,65,66,67,68]. Glaucoma donor tissue exhibits an elevated presence of microglia in the inner retina and optic nerve, as well as an increase in GFAP, indicating reactive restructuring of astrocytes and Müller glia [60,69,70]. 

The glial responses in glaucoma and their association with RGCs and systemic immunity have been most accurately characterized in animal models [50,71,72]. 

### 3.4. Autophagy

Autophagy contributes to the development of glaucoma. Cellular stress, such as hunger, hypoxia, and oxidative damage, triggers a significant reaction [73]. Nevertheless, numerous studies present contradictory information regarding whether this response is harmful or beneficial [74,75,76,77]. Among all conditions, a lack of nutrition (particularly amino acids) is the most important factor in autophagy activation. Autophagy levels decrease with the natural process of human aging in all investigated tissues, including the brain and retina. This drop plays a role in the development of several neurological illnesses, such as glaucoma [78]. 

### 3.5. Excitotoxicity

Given that glutamate is a highly toxic substance for the nervous system, glutamate transporters play a crucial role in preventing retinal damage from excessive neural stimulation. Excitotoxicity has been implicated in various eye diseases, such as retinal or choroidal artery occlusion-induced ischemia, glaucoma, and diabetic retinopathy. Considering the highly toxic nature of glutamate in the nervous system, the role of glutamate transporters is essential in reducing damage to the retina induced by excessive neuronal stimulation. Both experimental and clinical cases of glaucoma exhibit increased amounts of glutamate in the vitreous [79,80]. Excessive levels of glutamate cause an overactivation of N-Methyl-D-aspartate (NMDA) receptors, particularly impacting RGCs death [81,82,83,84].

### 3.6. Decreased Ocular Perfusion 

Numerous chronic neurodegenerative disorders have been linked to vascular factors [85]. The level of ocular perfusion is affected by the resistance to blood flow and determines the amount of blood and oxygen that reaches the optic nerve head [86]. Therefore, there is a generally held belief that a decrease in ocular perfusion pressure may heighten the susceptibility of the optic disc, resulting in an elevated likelihood of glaucoma onset or advancement [87,88,89,90].

## 4. Exploring the Frontiers of Neuroprotection Treatments

Currently, glaucoma is acknowledged as a long-lasting neurodegenerative illness that impacts the complete visual pathway extending from the eye to the visual cortex. It is a prominent factor contributing to decreased eyesight in the senior population worldwide, and its impact as a social health concern is expected to worsen over time. Given its neurodegenerative character, pharmacological treatments applied to several degenerative brain diseases could also help in treating glaucoma and other visual neuropathies [91]. Neuroprotection has gained significant interest in recent years as a new method to prevent or slow down the progression of structural and functional damage caused by glaucoma, with a specific emphasis on delaying the degeneration of RGCs [92] (Table 1).

### 4.1. Nitric Oxide

Nitric oxide (NO) makes trabecular meshwork (TM) cells relax and Schlemm’s canal (SC) cells more permeable. It has diverse biological functions in the ocular system, such as regulating vascular tone and performing several additional jobs [93,94], processing visual stimuli [95], promoting immunological cytotoxicity [96], and managing IOP [97,98]. Healthy individuals exhibit higher levels of NO production in comparison to ocular hypertensive patients [99]. Moreover, the existing literature indicates that NO has a dual role in optic neuropathy in cultured neurons and rat models. Low levels of NO have been found to have neuroprotective benefits, while high levels of NO have been associated with neurodegenerative consequences [62,100,101,102]. Vyzulta™ is a commercially available and licensed eye drop that comprises a solution of Latanoprost Bound at a concentration of 0.024%. This eye drop is a prostaglandin F2α analogue that donates NO. It is used to reduce IOP in patients with open-angle glaucoma or ocular hypertension [103].

### 4.2. Rho Kinase Inhibitor

As mentioned in Section 2.7, Rho kinase inhibitors are employed in the management of neurodegenerative disorders. They have been observed to prevent the programmed cell death of RGCs caused by axotomy [104], and also provide protection against the loss of neurons in vivo [105,106].

### 4.3. Coenzyme Q10

Coenzyme Q10 has been extensively used to treat a range of illnesses, such as Leber hereditary optic neuropathy, cerebral ischemia, Parkinson’s disease, and Huntington’s disease [107]. It has demonstrated efficacy in reducing apoptosis and the loss of RGCs in animal models. When administered directly into the eye, it prevented the death of RGCs through the regulation of mitochondrial depolarization [108,109]. 

### 4.4. Citicoline(Cytidine-5′-Diphosphocholine)

Citicoline is a naturally occurring chemical that plays a role in the production of phospholipids’ membranes, specifically phosphatidylcholine. The effects of citicoline are caused by a lot of different metabolic pathways, such as cholinergic and dopaminergic transmission, phospholipid homeostasis, and mitochondrial dynamics. These mechanisms play a role in the complex process of visual transmission [109,110]. Citicoline’s neuroprotective effect is due to its ability to decrease glutamate excitotoxicity [111] and oxidative stress [112], improve mitochondrial function [113], and enhance neurotrophin levels [114,115]. However, current research does not provide enough support to conclude that citicoline effectively slows down the course of glaucoma [115].

### 4.5. Gingko Biloba Extract (GBE)

Ginkgo is a prehistoric tree species that closely resembles plants that existed 270 million years ago. The cultivation of this tree is extensive in China, and it was introduced at an early stage in traditional Eastern medicine to address a range of issues, including asthma, vertigo, tiredness, tinnitus, and circulatory ailments. Current medical science has scientifically proven the positive effects of ginkgo biloba extract 761, derived from the leaves of the ginkgo biloba tree, on cognitive impairment and dementia. Because Alzheimer’s disease and glaucoma share molecular and mechanical similarities, researchers have investigated ginkgo for glaucoma [78]. Studies conducted on animal models have demonstrated that GBE possesses neuroprotective, antioxidant, and anti-inflammatory characteristics, specifically targeting RGCs [73,116,117,118]. It also demonstrated that GBE enhances the flow of blood in the eyes. However, the impact of GBE on the visual field remains uncertain [119,120].

### 4.6. Tumor Necrosis Factor-α Inhibitors

Tumor necrosis factor α (TNF-α) has been shown to be involved in pathways leading to the death of RGCs death in animal and in vitro glaucoma models [121]. It stimulates the processes that lead to the death of mitochondria cells and triggers the production of reactive oxygen species (ROS) [122], play a vital function in the immune response and are responsible for a apoptosis and inflammation [69] and potentially influence axonal degeneration in glaucomatous eyes as well [123]. It is feasible to repair damaged neuronal tissue caused by inflammation in glaucomatous eyes by blocking TNF, which is the key factor in both inflammatory and cell death signaling in RGCs, as well as axon injuries in in vivo models of glaucoma [124].

### 4.7. Nuclear Factor Kappa B (NF-κB) Inhibitors

The nuclear factor-kappaB (NF-κB) is a crucial molecule that activates the transcription of genes involved in inflammation. It has been found to be significantly impacted in astroglia damaged by glaucoma [125]. Research has demonstrated the crucial involvement of astroglial NF-κB in the inflammatory and degenerative effects of experimental glaucoma, and its ability to offer neuroprotection by modulating the immune response [126].

### 4.8. Memantine

Memantine can help treat Alzheimer’s disease and Parkinson’s disease [127,128,129]. It is a non-competitive open-channel blocker that only inhibits NMDA-receptor function when glutamate levels are abnormally high, leaving normal physiological levels necessary for synaptic action unaffected, and has been discovered to be very successful as a neuroprotective drug in both acute and chronic animal models of RGC death [130,131,132,133].

Regrettably, in two clinical trials, the administration of memantine on a daily basis for a duration of 4 years did not have any effect in terms of preventing or slowing down the progression of open-angle glaucoma in patients [134].

### 4.9. Calcium Channel Blockers (CCBs)

Calcium channel blockers (CCBs) are authorized for the management of hypertension and cerebral impairment. They enhance vasodilation, reduce vascular resistance, and ultimately lower blood pressure while increasing blood flow [135]. Studies have demonstrated that CCBs possess therapeutic efficacy in the treatment of glaucoma. Nifedipine [136], verapamil, diltiazem [137], and nilvadipine [138] have been found to decelerate the advancement of visual field deterioration in patients with normal tension glaucoma (NTG). Furthermore, the administration of nifedipine and brovincamine resulted in an augmentation of peripheral blood circulation in the optic disc, as indicated by previous studies [136,139].

### 4.10. Platelet-Derived Growth Factor (PDGF)

Platelet-derived growth factor (PDGF) plays a crucial role in the neuroprotective impact of mesenchymal stem cells on RGCs in both retinal explants and experimental glaucoma [140,141]. Additionally, it has been observed to increase neuronal survival in many damage models, such as excitotoxicity [142] and oxidative stress [143]. These findings recommend further exploration of PDGF treatment as a potential glaucoma therapy in the future.

### 4.11. Neurotrophic Factors 

Brain-derived neurotrophic factor (BDNF), one of the neurotrophin families, has significantly low levels in the ocular fluids found in glaucoma patients [78]. This supports the idea that a lack of neurotrophic factors is a probable cause of glaucomatous optic neuropathy [144]. Several published studies detail the connection between the absence of BDNF support for the RGCs and the initiation of their deaths [145,146,147]. After 7 days of elevated IOP in a rat model of glaucoma, the natural production of brain-derived BDNF and neurotrophin-4/5 (NT-4/5) in the optic nerve was entirely depleted, which corresponds to the apoptosis of RGCs. Essentially, a lack of neurotrophic factors in the optic nerve is a contributing factor to the development and advancement of glaucomatous optic neuropathy (GON). Augmenting neurotrophic support can potentially postpone RGC degeneration in individuals with glaucoma [40]. Thus, directing attention towards modifying the problematic sequence around the converging endpoints could potentially be more effective in preventing the death of RGCs [148].

### 4.12. Erythropoietin

Erythropoietin (EPO) is a hormone that can promote pleiotropic effects [149]. Multiple studies have documented an elevation in the EPO concentration in the fluid known as aqueous humor among individuals diagnosed with glaucoma [135]. The increasing levels of aqueous EPO in glaucomatous eyes may be attributed to ischemia, hypoxia, or increased levels of reactive oxygen species (ROS) resulting from glaucomatous damage [150]. EPO exhibits anti-inflammatory [151], antioxidative [151], and antiapoptotic effects [152], which make it a prospective therapeutic option for the treatment of glaucoma [153,154].

## 5. A Multifaceted Approach: The Future of Glaucoma Management

### 5.1. Nanotechnology-Based Ophthalmic Delivery System

Novel drug carriers, including nanomicelles, nanoparticles (NPs), nanoemulsions (NEs), microemulsions, nanofibers, dendrimers, liposomes, niosomes, nanowafers, and microneedles (MNs), have been investigated for the treatment of both anterior and posterior ocular disorders [155,156]. An instance of the application of lipid-based nanoparticles (NPs) containing brimonidine and latanoprost is their usage in the treatment of glaucoma [157,158].

### 5.2. Intracameral Implant

Bimatoprost implant (Durysta) is a biodegradable intracameral implant to reduce IOP in patients with open-angle glaucoma(OAG) or ocular hypertension (OHT) [159]. The implant is a tiny cylinder with a diameter of around 200 µm and a length of about 1.1 mm. It contains 10 µg of bimatoprost and uses a drug delivery system for the eyes. This system is made of biodegradable polymers that have been proven to be safe for ocular tissues [160]. The implant was specifically engineered to gradually release bimatoprost over a period of approximately 3–4 months. However, there have been reports that the implant has long-term effects on IOP. The most frequently reported adverse effect in patients was eye irritation. The FDA currently permits a single dose of the implant per eye through intracameral delivery, and it prohibits re-treatment [161].

The travoprost intracameral implant (iDose TR) is a small, biocompatible titanium reservoir that is secured to the sclera at the iridocorneal angle by an anchor that passes through the trabecular meshwork. The reservoir has 75 μg of a proprietary, preservative-free travoprost formulation, which is about 25,000 times more concentrated than the travoprost in travoprost ophthalmic solution [162]. For individuals with OHT or OAG, the FDA has approved a single injection per eye.

### 5.3. Genome-Wide Analyses

Prior genome-wide association studies have discovered significant loci linked to the incidence of POAG [163,164,165,166]. Identifying risk loci could be used as targets for preventive therapies.

### 5.4. Stem Cell Therapy

Stem cell therapy offers the intriguing potential to regenerate and replenish RGCs, potentially leading to the restoration of eyesight that has been lost due to glaucoma [2,167]. It could potentially contribute to the restoration of the trabecular meshwork using cell-based functional methods. Existing evidence indicates the presence of a population of mature stem cells in the Schwalbe’s ring and the anterior trabecular meshwork [168].

## 6. Conclusions

The comprehension of the intricate pathophysiology of glaucoma is expanding, leading to a transformation in the emphasis on therapeutic interventions. Prospects for the future indicate the convergence of IOP-lowering, neuroprotection, and cellular resilience-building strategies into a unified approach that provides holistic management solutions and strives for individuality, precision, and efficacy to predominate. As research progresses, there is an increasing focus on the genetic and molecular underpinnings of the disease, which holds promise for the development of personalized medicine tailored to each patient’s unique genetic profile. Furthermore, advances in imaging technologies and biomarker discovery are enhancing early detection and monitoring capabilities, allowing for more timely and targeted interventions. Ultimately, these advancements aim to preserve vision and improve the quality of life for patients with glaucoma, marking a significant shift towards more comprehensive and patient-centered care in ophthalmology.

## Figures and Tables

**Table 1:** Neuroprotective treatments.

Pathological Mechanism	Result or Impact	Neuroprotective Interventions
Deprivation of Neurotrophins	Glutamate NMDA Receptors Activation, Calcium Influx of RGCs	Neurotrophins
Glutamate Increase	Increased activation of NMDA receptors	Coenzyme Q10, Memantine
Glutamate NMDA Receptors Activation	Leads to calcium influx into RGCs	Brimonidine, BDNF-TrkB signaling
Calcium Influx of RGCs	Leads to an increase in free radicals (e.g., NO), Mitochondrial Dysfunction	Calcium channel blockers
Oxidative Stress	Leads to RGCs death	Antioxidant (e.g., EPO), NOS inhibitors
Decreased Ocular Perfusion	Leads to RGCs death	Gingko biloba extract
Neuroinflammation	Leads to RGCs death	
Mitochondrial Dysfunction	Leads to excitotoxicity and RGCs death	Citicoline
RGCs Death	Final outcome	
